# Assessing glomerular filtration rate (GFR) in critically ill patients with acute kidney injury - true GFR versus urinary creatinine clearance and estimating equations

**DOI:** 10.1186/cc12777

**Published:** 2013-06-15

**Authors:** Gudrun Bragadottir, Bengt Redfors, Sven-Erik Ricksten

**Affiliations:** 1 Department of Anaesthesiology and Intensive Care Medicine, Sahlgrenska Academy, University of Gothenburg, Sahlgrenska University Hospital, Gothenburg, Sweden

## Abstract

**Introduction:**

Estimation of kidney function in critically ill patients with acute kidney injury (AKI), is important for appropriate dosing of drugs and adjustment of therapeutic strategies, but challenging due to fluctuations in kidney function, creatinine metabolism and fluid balance. Data on the agreement between estimating and gold standard methods to assess glomerular filtration rate (GFR) in early AKI are lacking. We evaluated the agreement of urinary creatinine clearance (CrCl) and three commonly used estimating equations, the Cockcroft Gault (CG), the Modification of Diet in Renal Disease (MDRD) and the Chronic Kidney Disease Epidemiology Collaboration (CKD-EPI) equations, in comparison to GFR measured by the infusion clearance of chromium-ethylenediaminetetraacetic acid (^51^Cr-EDTA), in critically ill patients with early AKI after complicated cardiac surgery.

**Methods:**

Thirty patients with early AKI were studied in the intensive care unit, 2 to 12 days after complicated cardiac surgery. The infusion clearance for ^51^Cr-EDTA obtained as a measure of GFR (GFR_51Cr-EDTA_) was calculated from the formula: GFR (mL/min/1.73m^2^) = (^51^Cr-EDTA infusion rate × 1.73)/(arterial ^51^Cr-EDTA × body surface area) and compared with the urinary CrCl and the estimated GFR (eGFR) from the three estimating equations. Urine was collected in two 30-minute periods to measure urine flow and urine creatinine. Urinary CrCl was calculated from the formula: CrCl (mL/min/1.73m^2^) = (urine volume × urine creatinine × 1.73)/(serum creatinine × 30 min × body surface area).

**Results:**

The within-group error was lower for GFR_51Cr-EDTA _than the urinary CrCl method, 7.2% versus 55.0%. The between-method bias was 2.6, 11.6, 11.1 and 7.39 ml/min for eGFR_CrCl_, eGFR_MDRD_, eGFR_CKD-EPI _and eGFR_CG_, respectively, when compared to GFR_51Cr-EDTA_. The error was 103%, 68.7%, 67.7% and 68.0% for eGFR_CrCl_, eGFR_MDRD_, eGFR_CKD-EPI _and eGFR_CG_, respectively, when compared to GFR_51Cr-EDTA_.

**Conclusions:**

The study demonstrated poor precision of the commonly utilized urinary CrCl method for assessment of GFR in critically ill patients with early AKI, suggesting that this should not be used as a reference method when validating new methods for assessing kidney function in this patient population. The commonly used estimating equations perform poorly when estimating GFR, with high biases and unacceptably high errors.

## Introduction

Accurate assessment of kidney function in the critically ill patient plays an important role in diagnosing acute kidney injury (AKI); in the appropriate prescription and dosing of drugs, whose elimination depends on renal function; and in timely application of therapeutic strategies. In clinical medicine, the concentration of creatinine in serum is used daily as a marker for kidney function. However, serum creatinine concentration may not be suitable for this purpose as it is affected by factors other than kidney function. Creatinine is metabolized from creatine, which is released by the muscles, therefore muscle mass and metabolic transformation of creatine have an impact on serum creatinine concentration [1]. In addition, age, gender and race all affect muscle mass and, in turn, serum creatinine concentrations [2,3].

In critically ill patients with AKI, three main factors influence the accuracy of serum creatinine as a marker of kidney function: true kidney function, fluctuations in creatinine production, and fluid balance. In critically ill patients, creatinine production may be decreased because of immobilization and malnutrition, or increased because of catabolic illness. Increases in total body water, common in these patients, increases the distribution volume of creatinine, and attenuates the increase in serum creatinine concentration caused by AKI [1,4-6]. Furthermore, various drugs used to treat critically ill patients, such as cimetidine and trimethoprim-sulfamethoxazole, are known to compete with the active tubular secretion of creatinine, and therefore to affect serum creatinine concentration [7,8]. Thus, daily changes in serum creatinine poorly reflect changes in kidney function in patients with AKI [4].

Glomerular filtration rate (GFR), measured using exogenous substances such as inulin, iohexol, ^123^I-iothalamate, diethylene triamine pentaacetic acid and chromium-ethylenediaminetetraacetic acid (^51^Cr-EDTA) as filtration markers, is considered as the gold standard for assessment of renal function [9,10]. Unfortunately, measuring GFR with these markers is expensive and complex, considerably outweighing their high reliability and making them unsuitable for routine use in the intensive care setting.

The second best method for assessment of renal function is urinary creatinine clearance (CrCl), which can be computed from a timed urine collection (for example, a 24-hour urine collection) and blood sampling for serum creatinine [11]. However, clearance methods require a steady state situation, a criteria not always met in critically ill patients, where changes in the hemodynamic status can result in dramatic changes in renal function over a 24-hour urine collection period. Furthermore, accurate timed collection of urine is cumbersome, and the main source of error [12]. Finally, urinary CrCl may considerably overestimate GFR because of tubular secretion of creatinine [1,5].

GFR can also be assessed using estimating equations. These equations include variables such as age, sex, race and body weight, in addition to serum creatinine, as a substitute for muscle mass and they can therefore overcome some of the limitations associated with using serum creatinine alone [9,13-15]. Estimating equations for GFR have been developed in study populations consisting of patients with chronic stable kidney disease and stable serum creatinine concentrations [13-15]. These equations are poorly evaluated in critically ill patients with AKI and most often they have been validated against urinary CrCl, instead of a gold standard reference method, such as measurement of GFR using an exogenous substance for a filtration marker. Furthermore, data on the agreement between urinary CrCl and gold standard GFR in critically ill patients with early AKI are lacking.

The aim of this study was to evaluate the agreement [16] of urinary CrCl and three commonly used estimation equations, the Cockcroft-Gault (CG) [13], Modification of Diet in Renal Disease (MDRD) [14,17,18] and Chronic Kidney Disease Epidemiology Collaboration (CKD-EPI) [15] equations, for estimating GFR in comparison to GFR measured by the infusion clearance of ^51^Cr-EDTA, in critically ill patients with early AKI after complicated cardiac surgery.

## Methods

### Study population

The study protocol was approved by the Human Ethics Committee of the University of Gothenburg. Informed consent was obtained from the patient or, if the patient was sedated, the patient's next of kin, before enrolment in the study. Thirty patients who developed AKI after complicated heart surgery were included in the study according to the following inclusion criteria: cardiac surgery with cardiopulmonary bypass; normal preoperative renal function (serum creatinine ≤105 μmol/L); and development of early AKI according to the Acute Kidney Injury Network criteria, defined as a 50% to 300% postoperative increase in serum creatinine from baseline [19]. The following exclusion criteria were used: heart transplantation; thoraco-abdominal aortic surgery; aortic dissection; use of nephrotoxic drugs, such as radiocontrast agents, aminoglycoside antibiotics or nonsteroidal anti-inflammatory analgesics; or need of dialysis.

In the intensive care unit (ICU), patients who were mechanically ventilated were sedated with propofol. Morphine or fentanyl was used for treatment of postoperative pain. The hemodynamic and renal management of patients were at the discretion of the attending intensive care physician. The treatment protocol included inotropic support with milrinone, dopamine and/or norepinephrine to maintain a cardiac index ≥2.1 L/min/m^2^, whole body oxygen extraction ≤40%, and mean arterial pressure at 70 to 80 mmHg with or without an intra-aortic balloon pump. A continuous infusion of furosemid (5 to 40 mg/h) was used, if needed, to promote diuresis. When the patient was sedated, neurological status was not included in the sequential organ failure assessment score [20].

### Systemic hemodynamics

Arterial blood pressure was measured by a radial or femoral arterial catheter. All the patients had a central venous catheter. Systemic hemodynamics were measured by a pulmonary artery thermodilution catheter (Baxter Healthcare Corporation, Irvine, CA, USA) in 21 out of 30 patients. Measurements of thermodilution cardiac output were performed in triplicate. The pulmonary artery wedge pressure was measured intermittently. Systemic vascular resistance was calculated according to standard formula.

### Measurment of serum and urinary creatinine

Blood samples for serum creatinine were taken within a couple of hours before the experimental procedure. All serum and urinary creatinine measurements were performed in the same laboratory. Serum and urinary creatinine concentrations were analyzed by a standardized enzymatic colorimetric method using the Modular P clinical chemistry analyzer **(**Roche Diagnostic Scandinavia AB, Bromma, Sweden)

### Assessment of glomerular filtration rate

#### ^51^Cr-EDTA clearance and urinary creatinine clearance

After blood and urine blanks were taken, an intravenous priming dose of ^51^Cr-EDTA (0.6 MBq/m^2 ^body surface area) was given, followed by an infusion at a constant rate individualized to body weight and serum creatinine. Serum ^51^Cr-EDTA activities from arterial blood were measured by a well counter (Wizard 300, 1480, Automatic Gamma Counter, PerkinElmer Inc., Turku, Finland). After an equilibration period of at least 60 minutes, urine was collected in two 30-minute periods to measure urine flow and urine creatinine (period A and period B). An indwelling Foley catheter drained the urinary bladder. The levels of ^51^Cr-EDTA were obtained from arterial blood at the end of each urine collection period. Clearance for ^51^Cr-EDTA was obtained as a measure of GFR calculated from the formula:

$$\text{GFR}\left(\text{mL}/\text{min}/\text{1}.{\text{73m}}^{\text{2}}\right)=\;\left({}^{\text{51}}\text{Cr}-\text{EDTA infusion rate }\times \text{ 1}.\text{73}\right)\;/\;\left({\text{arterial}}^{\text{51}}\text{Cr}-\text{EDTA }\times \text{ body surface area}\right)$$

The mean of the two ^51^Cr-EDTA clearances (period A and B) was used for subsequent comparison with the urinary CrCl and the estimating equations. Urinary CrCl was calculated for period A and B from the formula:

$$\text{CrCl }\left(\text{mL}/\text{min}/\text{1}.\text{73}{\text{m}}^{\text{2}}\right)=\left(\text{urine volume }\times \text{ urine creatinine }\times \text{ 1}.\text{73}\right)/)\left(\text{serum creatinine }\times \text{ 3}0\text{ minutes }\times \text{ body surface area}\right)$$

#### Estimating equations

GFR was estimated in all patients by the use of three frequently used equations: the CG equation [13], the simplified refitted MDRD equation [18], and the CKD-EPI equation [15] (Additional file 1). The CG equation was calculated with the actual body weight, the preoperative body weight (to correct for the weight increase due to edema) and the preoperative ideal body weight (to correct for overweight), calculated according to a standard formula (Additional file 1). To allow comparison to the results of other estimating equations, the estimated GFR (eGFR) from the CG equation was normalized to body surface area = 1.73 m^2^. All the equations used for eGFR are summarized in Additional file 1.

### Statistical analysis

Data on hemodynamic and renal variables from periods A and B were compared using a paired t-test. A probability level (*P*-value) of less than 0.05 was considered to indicate statistical significance. The data are presented as mean ± standard error of the mean (mean ± SEM). Descriptive data analyses on the ^51^Cr-EDTA clearance method and the urinary CrCl method for measurement and assessment of GFR were performed according to Bland and Altman [16]. The (within-method) repeatability of each of these two methods were assessed by the error (double standard deviation of the absolute differences divided by the mean of the repeated measurements), the repeatability coefficient (the double standard deviation of the absolute differences) and the mean coefficient of variation (standard deviation of the mean divided by the mean of the repeated measurements).

The agreements between the gold standard ^51^Cr-EDTA infusion clearance method and the urinary CrCl method, as well as the estimating equations used for eGFR (the CG equation, the MDRD equation and the CKD-EPI equation), were assessed according to Bland and Altman [16]. The mean difference between two methods (bias) and the standard deviation of the differences were calculated as well as the error (double standard deviation divided by the mean of the measurements from the two methods) and the limits of agreement (mean difference ± two standard deviations). According to Critchley and Critchley, an acceptable within-method error was defined as 20% or less and between-method error as 30% or less [21].

## Results

Thirty patients were included in the study, 1 to 12 days after cardiac surgery. Baseline characteristics of the patients are presented in Table 1. In the ICU, 23 patients (77%) were sedated with propofol (52 ±4.6 μg/kg/min) and mechanically ventilated to normocapnia. Seven patients (23%) were unsedated and spontaneously breathing. The serum creatinine increased from a preoperative value of 87 ±3 μmol/L to 172 ±9 μmol/L, corresponding to a mean relative increase of 99 ±8% (range: 52% to 245%) on the day of the study. The patients had a mean sequential organ failure assessment score of 8.6 ±0.38 (range: 5 to 13). Twenty-eight patients (93%) were treated with norepinephrine infusion. Eighteen patients (60%) were treated with milrinone, two (7%) with dopamine and twenty-seven patients (90%) had furosemide infusion. Six patients (20%) needed an intra-aortic balloon pump on the day of study. Four patients (13%) needed dialysis later in the ICU (Table 2). The 30-day postoperative mortality was 20% in this group of patients.

**Table 1:** Baseline characteristics

Preoperative characteristics	
Gender, n (% men)	24 (80%)
Age (year)	68.0 ±1.71
Body weight (kg)	87.4 ±2.95
Body surface area (m^2^)	2.0 ±0.04
Preoperative left ventricular ejection fraction (%)	43 ±3
Diabetes type 2, n (%)	7 (23%)
Hypertension, n (%)	15 (50%)
Preoperative serum creatinine (μmol/L)	86.5 ±3.00
Preoperative Higgins risk score *n *= 19	3.6 ±0.46
Preoperative treatment:	
Angiotensin-converting enzyme inhibitor, n (%)	18 (60%)
Beta-adrenergic blocker, n (%)	24 (80%)
Calcium antagonists, n (%)	3 (10%)

**Perioperative characteristics**	

Type of surgery	
Coronary artery bypass surgery, n (%)	12 (40%)
Valve, n (%)	5 (17%))
Combined, n (%)	7 (23%)
Other, n (%)	4 (13%)
Redo CABG/Valve	2 (7%)
Nonelective^a^, n (%)	9 (30%)
Cardiopulmonary bypasstime (min)	145.9 ± 12.65
Aortic cross clamp time (min)	81.8 ± 8.88
Intensive care unit Higgins risk score	9.1 ± 0.93
Postoperative (day 1) serum creatinine (μmol/L)	119.2 ±5.59

Data are presented as means ± SEM. ^a ^Surgery performed within 24 hours after referral.

**Table 2:** Individual data at inclusion to the study

Patient number	Study entry (day)	Preoperative creatinine (μmol/L)	Inclusion creatinine (μmol/L)	Creatinine increase (%)	AKIN stage	SOFA Score	IABP	Norepinephrine (μg/kg/min)	Milrinone (μg/kg/min)	Dopamine (μg/kg/min)	Furosemid (μg/kg/min)	CRRT in ICU
1	5	65	136	109	2	9	No	0.25	0.00	0	2.53	No
2	2	107	209	95	1	10	Yes	0.16	0.24	0	1.02	No
3	3	109	200	83	1	8	Yes	0.12	0.13	0	0.80	No
4	4	91	151	66	1	12	No	0.14	0.18	0	0.00	No
5	4	102	170	67	1	7	No	0.09	0.00	0	0.99	No
6	6	78	145	86	1	9	No	0.43	0.43	0	0.00	No
7	6	101	194	92	1	7	No	0.22	0.00	0	2.22	No
8	1.5	90	146	62	1	10	Yes	0.33	0.44	0	3.70	No
9	3	81	230	184	2	10	No	0.11	0.52	0	3.06	No
10	6	93	210	126	2	7	No	0.32	0.25	0	1.05	No
11	5	84	217	158	2	9	No	0.27	0.00	0	0.95	No
12	1.5	82	135	65	1	10	No	0.33	0.26	0	3.21	No
13	2	102	155	52	1	10	Yes	0.92	0.26	0	7.41	No
14	4	83	182	119	2	10	No	0.95	0.20	0	6.53	Yes
15	2	81	127	57	1	6	Yes	0.39	0.40	0	2.22	No
16	2	129	233	81	1	11	No	0.54	0.30	2	7.58	No
17	2	79	131	66	1	8	No	0.10	0.00	0	3.06	No
18	1	80	150	88	1	7	No	0.10	0.00	0	4.76	No
19	4	62	134	116	2	10	Yes	0.21	0.50	0	1.14	Yes
20	6	105	184	75	1	4	No	0.00	0.25	0	0.00	No
21	8	105	362	245	3	9	No	0.02	0.35	2	5.34	Yes
22	12	84	187	123	2	9	No	0.05	0.00	0	1.75	No
23	12	63	108	71	1	8	No	0.47	0.00	0	4.14	No
24	1	67	127	90	1	8	No	0.92	0.50	0	3.47	Yes
25	3	69	162	135	2	6	No	0.02	0	0	6.67	No
26	5	79	163	106	2	8	No	0.40	0	0	5.55	No
27	6	64	119	86	1	7	No	0.15	0	0	4.63	No
28	4	99	156	58	1	13	No	0.40	0.50	0	9.44	No
29	8	93	156	68	1	5	No	0	0	0	2.53	No
30	8	69	173	151	2	12	No	0.55	0.20	0	8.58	No
Mean	4.6 ±0.53	86.5 ±3.0	171.7 ±8.96	99.3 ±7.81		8.6 ±0.38		0.32 ±0.05^a^	0.33 ±0.03^a^	2.0 ±0.00^a^	3.86 ±0.49^a^	

^a^Mean and SEM among treated. AKIN, Acute Kidney Injury Network; CRRT, chronic renal replacement therapy; IABP, intraaortic balloon pump; SOFA, sequential organ-failure assessment.

Individual data on GFR and the various eGFR:s (CrCl, MDRD, CKD-EPI and CG) are shown in Table 3. Hemodynamic and renal data obtained during measurement periods A and B are presented in Table 4. With the exception of heart rate (*P *= 0.03) there was no statistically significant differences in hemodynamic data, plasma ^51^Cr-EDTA concentrations, GFR measured by ^51^Cr-EDTA infusion clearance (GFR_51Cr-EDTA _) and eGFR measured by CrCl (eGFR_CrCl_) between periods A and B.

**Table 3:** Individual data on measured and estimated glomerular filtration rates (mL/min/1.73m^2^) in 30 critically ill patients

Patient	^51^Cr-EDTA	CrCl	MDRD	CKD-EPI		CG	
					
					actual body weight	preop body weight	ideal body weight
1	39	39	34	35	37	37	34
2	40	31	27	27	29	29	27
3	41	31	29	28	34	35	28
4	76	37	40	40	36	36	39
5	26	49	35	36	50	51	36
6	56	23	41	41	34	34	38
7	42	34	31	31	34	34	34
8	45	46	31	32	39	39	33
9	41	48	25	24	31	30	26
10	27	28	20	19	23	23	19
11	41	29	27	27	31	31	29
12	74	46	46	47	54	54	46
13	47	38	40	41	47	49	42
14	49	12	32	31	35	33	30
15	76	108	50	53	53	53	52
16	36	16	23	22	23	23	21
17	88	57	47	49	55	55	48
18	78	75	40	41	47	47	38
19	40	80	35	36	44	43	45
20	45	43	32	32	35	36	33
21	27	15	14	13	16	16	15
22	33	43	22	21	24	24	21
23	74	43	62	71	72	72	67
24	60	64	49	49	50	50	46
25	35	33	26	26	35	35	23
26	31	71	37	38	45	44	40
27	29	58	51	49	47	46	40
28	28	31	40	42	43	40	45
29	27	70	38	38	36	37	35
30	60	34	39	42	50	52	48

**Mean ±SEM**	47 ±3	44 ±3	35 ±2	36 ±2	40 ±2	40 ±2	36 ±2

^51^Cr-EDTA, glomerular filtration rate measured with infusion clearance of ^51^Cr-EDTA; CG, estimated glomerular filtration rate with the Cockcroft Gault equation using actual, preoperative (preop) and preoperative ideal body weight; CKD-EPI, estimated glomerular filtration rate with the chronic Kidney Disease Epidemiology Collaboration equation; CrCl, estimated glomerular filtration rate measured with urinary creatinine clearance; MDRD, estimated glomerular filtration rate with the Modification of Diet in Renal Disease equation.

**Table 4:** Hemodynamic and renal data for periods A and B

	Period A	Period B	*P*
Mean arterial pressure (mmHg)	75.6 ±1.29	73.8 ±1.13	0.10
Cardiac output (L/min), *n *= 21	5.8 ±0.30	5.8 ±0.31	0.77
Heart rate (beats/min)	94.6 ±3.95	91.2 ±3.22	0.03^a^
Central venous pressure (mmHg)	11.6 ±0.69	11.8 ±0.76	0.46
Systemic vascular resistance (dynes × sec × cm^-5^), *n *= 21	905.3 ±46.0	897.7 ±56.2	0.71
Pulmonary capillary wedge pressure (mmHg) *n *= 21	16.5 ±1.39	16.3 ±1.34	0.69
Diures (mL/min)	3.87 ±0.32	3.80 ±0.34	0.67
Plasma ^51^Cr-EDTA	325.0 ±21.5	328.2 ±21.46	0.06
Measured glomerular filtration rate (^51^Cr-EDTA) (mL/min)	47.3 ±3.37	46.7 ±3.26	0.06
Measured urinary creatinine clearance (mL/min)	45.5 ±4.21	42.7 ±3.91	0.13

Data are presented as means ± SEM. ^a ^*P *<0.05. ^51^Cr-EDTA, chromium ethylenediaminetetraacetic acid.

### Repeatability within methods

**Figure 1:**
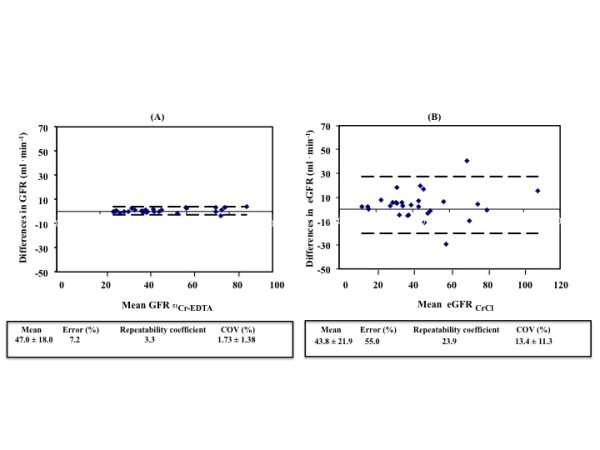
**Repeated measurements of glomerular filtration rate**. **(A) **^51^Cr-EDTA infusion clearance method and **(B) **the urinary CrCl method. The within group error, the repeatability coefficient and the coefficient of variation, is lower for GFR_51Cr-EDTA _than eGFR_CrCl_. COV, coefficient of variation; eGFR; estimated GFR; GFR, glomerular filtration rate; GFR_51Cr-EDTA_, GFR measured with ^51^Cr-EDTA infusion clearance; GFR_CrCl_, GFR estimated with urinary creatinine clearance.

As shown in Figure 1, the mean value for GFR_51Cr-EDTA _was 47.0 ±18.0 ml/min/1.73m^2 ^and for eGFR_CrCl _was 43.8 ±21.9 ml/min/1.73m^2^. The within-group error was lower for GFR_51Cr-EDTA _than eGFR_CrCl_, 7.2% versus 55.0%, respectively. The repeatability coefficient for GFR_51Cr-EDTA _and eGFR_CrCl _were 3.3 and 23.9, respectively, and the mean coefficient of variation for GFR_51Cr-EDTA _was lower than the mean coefficient of variation for eGFR_CrCl_, 1.73 ±1.38% versus 13.4 ±11.3%.

### Agreement between methods

**Figure 2:**
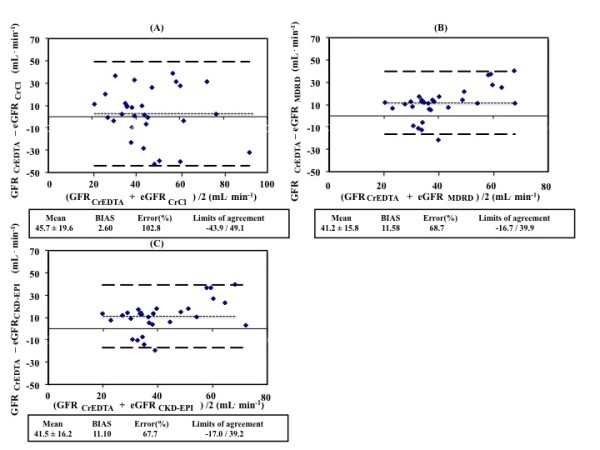
**Agreement between measured glomerular filtration rate and estimations from equations**. **(A) **GFR_51Cr-EDTA _and eGFR_CrCl_; **(B) **GFR _51Cr-EDTA _and eGFR_MDRD_; **(C) **GFR _51Cr-EDTA _and eGFR_CKD-EPI_. GFR, glomerular filtration rate; GFR_51Cr-EDTA_, GFR measured with ^51^Cr-EDTA infusion clearance; GFR_CG_, GFR estimated with the Cockcroft Gault equation; GFR_CKD-EPI_, GFR estimated with Chronic Kidney Disease Epidemiology Collaboration equation; GFR_CrCl_, GFR estimated with urinary creatinine clearance; GFR_MDRD_, GFR estimated with the Modification of Diet in Renal Disease equation.

**Figure 3:**
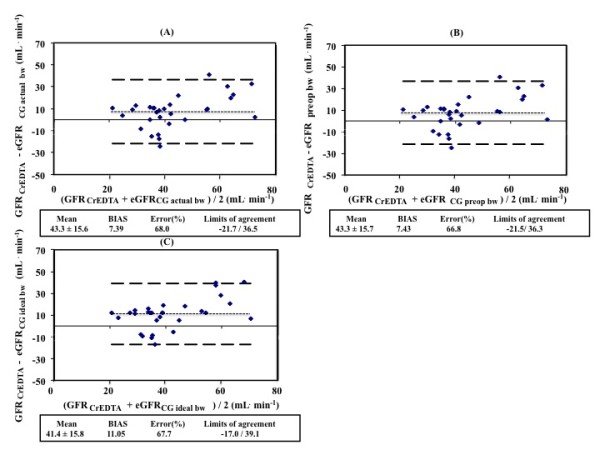
**Agreement between measured glomerular filtration rate and estimated rate caluclated using different weights in the CG equation**. **(A) **GFR_51 Cr-EDTA _and eGFR_CG actual bw_; **(B) **GFR_51 Cr-EDTA _and eGFR_CG __preop bw_; **(C) **GFR_51 Cr-EDTA _and eGFR_CG __ideal __bw_. bw, body weight; GFR, glomerular filtration rate; GFR_51Cr-EDTA_, GFR measured with ^51^Cr-EDTA infusion clearance; GFR_CG_, GFR estimated with the Cockcroft Gault equation.

The agreement between measured GFR (GFR_51Cr-EDTA_) and estimated GFR (eGFR) with the urinary CrCL method and the prediction equations (eGFR_CG_, eGFR_MDRD_, eGFR_CKD-EPI_) are described in Figures 2 and 3. When compared to GFR_51Cr-EDTA_, the between-method bias was 2.6 ml/min for eGFR_CrCl_, 11.6 ml/min for eGFR_MDRD _and 11.1 ml/min for eGFR_CKD-EPI_. The between-method bias was 7.39 ml/min for eGFR_CG _using actual body weight, 7.43 ml/min for eGFR_CG _using preoperative body weight and 11.1 ml/min for eGFR_CG _using preoperative ideal body weight when compared to GFR_51Cr-EDTA_.

The error was 103%, 68.7% and 67.7% for eGFR_CrCl_, eGFR_MDRD _and eGFR_CKD-EPI_, respectively, when compared to GFR_51Cr-EDTA _(Figure 3). The limits of agreement were -43.9 to 49.1, -16.7 to 39.9 and -17.0 to 39.2 ml/min, respectively, when compared to GFR_51Cr-EDTA_. The error was 68.0%, 66.8% and 67.7%, respectively, and the limits of agreement were -21.7 to 36.5, -21.5 to 36.3 and -17.0 to 39.1 ml/min for eGFR_CG _using actual body weight, eGFR_CG _using preoperative body weight and eGFR_CG _using preoperative ideal body weight when compared to GFR_51Cr-EDTA_.

## Discussion

In the present study on critically ill patients with early AKI, the performance of urinary CrCl and various estimation equations for assessment of GFR were compared to one of the gold standard techniques for measurement of GFR, the ^51^Cr-EDTA infusion clearance technique. The main findings were that urinary CrCl, which has been used as a reference method, had an unacceptably low repeatability and that all methods showed a poor agreement with the gold standard technique and can therefore not be considered as reliable methods to assess GFR in critically ill patients with early AKI.

Although commonly used in the ICU, comparisons of the urinary CrCl method for assessing GFR with gold standard GFR measurements have not, to our knowledge, previously been reported in critically ill patients with early AKI. Robert *et al*. compared urinary CrCl to inulin clearance in 20 mechanically ventilated, hemodynamically stable patients not requiring inotropic support, a minority of whom had acute renal dysfunction [22]. They found that there was a poor correlation between both 30-minute and 24-hour urinary CrCl and inulin clearance. However, agreement between the two methods was not tested according to Bland and Altman [16]. Erley *et al*. validated 24-hour urinary CrCl to inulin clearance in 31 ICU patients with a stable (three days) but wide range of renal (dys)function (serum creatinine: 53 to 590 μmol/l) [12]. Although they did not calculate the bias or error, they found a mean ratio of CrCl over inulin clearance of 1.03 with a 95% confidence interval between 0.54 and 1.92, suggesting a low bias but a high error, that is, 95% of the CrCl values could be up to 92% higher and 44% lower than the inulin clearance values. The results of the present study on early AKI are in line with the data from Erley *et al. *[12].

In a study of method comparison, assessment of within-method repeatability is important, because the repeatability of each of two methods limits the amount of agreement, which is possible [16,23]. Clearly defined criteria for an acceptable agreement between two methods have been lacking since the publication of Bland and Altman [16]. In an attempt to clarify the criteria for acceptable agreement between two methods, Critchley and Critchley suggested that acceptance of a new method should relay on a between-method error of up to 30% [21]. They could also demonstrate that the limits of within-group error of both the test and the reference method should be 20% or less to achieve a between-group error of 30% or less. In this study, the repeatability for ^51^Cr-EDTA clearance was high, with a within-method error of only 7.2%. However, urinary CrCl had an unacceptably low repeatability, with a within-method error of 55%. It is therefore not surprising that the agreement between the ^51^Cr-EDTA clearance and urinary CrCl was very low, with an unacceptably high between-method error of 103%.

The main source of error in the case of urinary CrCl, as demonstrated by Erley *et al. *[12] and by the present study, is probably the collection of urine, despite the fact that bladder catheters were used for urine collection. We have previously compared the within-method error of the urinary clearance of ^51^Cr-EDTA to that of the infusion clearance of ^51^Cr-EDTA measured simultaneously in ICU patients [24]. The latter method, which was used in the present study, does not require urine sampling but requires an equilibrium between the rate of infusion and excretion of the filtration marker. It was shown that the within-method error was 33% for the urinary clearance and 11% for the infusion clearance of ^51^Cr-EDTA [24]. This illustrates the inherent limitations of urinary clearance methods for the assessment of GFR, irrespective of the filtration marker used.

Urinary CrCl may grossly overestimate GFR due to creatinine secretion at the tubular level. The magnitude of this overestimation increases as GFR declines, and may be as great as 141% for patients with GFR of <40 mL/min/1.73m^2 ^[25]. Robert *et al. *[22] compared urinary CrCl to inulin clearance and showed that urinary CrCl overpredicted GFR when GFR was <40 ml/min, but underpredicted GFR when GFR was >40 ml/min. In the present study, the mean GFR, assessed by ^51^Cr-EDTA clearance, was approximately 45 to 50 ml/min, which could explain the low bias (2.6 ml/min) comparing urinary CrCl to ^51^Cr-EDTA clearance.

Disparities between measured GFR and urinary CrCl in critically ill patients may result from several factors. Clearance methods require a steady state situation, a criteria not always met in critically ill patients. Variations in urine output values, due to changes in hormonal regulation of renal perfusion, changes in systemic hemodynamics, and alterations in creatinine production, secretion and metabolism, secondary to rapidly evolving underlying disease states, influence the accuracy of urinary CrCl in critically ill patients [22]. Inaccurate 24-hour urine collection is also a major pitfall in the determination of CrCl. A majority of comparative studies conducted in critically ill patients have used 24-hour CrCl as a reference method [22,26-30]. Shorter timed urine collection for calculating urinary CrCl is now proposed to improve clinical utility and diminish procedural error. Instead of 24-hour urine collection period, we used two 30-minute urine collection periods. Previous studies on critically ill patients have demonstrated that urinary CrCl calculated from shorter urine collection periods show good correlation with those values calculated from longer urine collection periods [22,26,31-33].

All the GFR estimating equations used in this study, the CG, MDRD and CKD-EPI equations, performed poorly when compared to infusion clearance of ^51^Cr-EDTA in this group of critically ill patients with early AKI. The biases ranged from 7.39 ml/min (eGFR_CG actual bw_) to 11.58 ml/min (eGFR_MDRD_). The between-group errors were unacceptably large, ranging from 66.8% to 68.7%, with wide limits of agreement for all the equations. The poor performance of the estimating equations in critically ill patients with early AKI may be explained in part by the methods and populations used to develop these equations. All equations were developed and validated in populations of non-ICU patients with chronic kidney dysfunction. The CG equation was originally designed to estimate 24-hour CrCl, and not GFR, in hospitalized patients with mild renal dysfunction [13]. The MDRD equation was developed using urinary ^125^I-iothalamate clearance as a reference in 1,628 patients with chronic kidney disease [14], and the CKD-EPI equation was developed using data from 8,254 people with and without chronic kidney failure, using iothalamate clearance as a reference [15].

Another explanation for the poor performance of the estimating equations is depressed production of creatinine, caused by rapid muscle loss, in ICU patients. Hoste *et al. *[34] studied recently admitted critically ill patients with serum creatinine levels within the normal range and found that 25% of these patients had a urinary CrCl below 60 ml/min/1.73m^2^. Urinary creatinine excretion was low in patients with low CrCl, suggesting a pronounced muscle loss and depressed production of creatinine. This could explain why the estimating equations, based on serum creatinine, overestimated GFR in the present study.

Estimating equations are also limited by the use of serum creatinine as a filtration marker. Accurate estimation of GFR from the serum level of creatinine requires a steady state. A rise in serum creatinine levels is observed only after significant loss of kidney function. Thus, serum creatinine concentrations lag behind the decline and recovery in glomerular filtration rate and is affected by factors other than kidney function, as discussed above for estimating GFR with the urinary CrCl method in critically ill patients with AKI. Thus, during non-steady state conditions, using creatinine-based equations to estimate GFR results in inaccurate assessment of kidney function [1,22,35].

The estimating equations used in this study, have not been validated in critically ill patients with early AKI. Two studies have compared the CG formula to gold standard reference method in critically ill patients. In the study by Robert *et al. *[22], the performance of the CG equation was compared to inulin clearance in 20 critically ill patients not needing inotropic support. They found that there was a good correlation between inulin clearance and the CG equation, using the ideal body weight, and that CG equation can better predict GFR than urinary CrCl. However, the precision of the CG equation to predict GFR, that is, the error, was not estimated. In the second study, Erley *et al. *[12] compared the CG formula to inulin clearance in 31 critically ill patients with stable renal function for at least 3 days before inclusion, and found that the results of the CG formula were not sufficiently accurate to predict GFR. The third estimating equation used in this study (CKD-EPI) has, to our knowledge, not been evaluated against true measures of GFR in any population of critically ill patients.

An issue regarding the use of the CG equation, is which patient weight should be used (actual, preoperative or preoperative ideal body weight), because creatinine generation is a function of muscle mass, not body mass. Recalculating the CG equation with the preoperative body weight and the preoperative ideal body weight, instead of using the actual weight of the patient, did not improve the agreement between the CG equation and the GFR_51Cr-EDTA_.

## Conclusions

The commonly utilized urinary CrCl method for assessment of GFR in critically ill patients with early AKI shows poor precision. Therefore, it should not be used as a reference method when validating new methods for assessing kidney function in this particular patient population. Furthermore, the commonly used estimating equations (CG, MDRD and CKD-EPI equations) perform poorly when estimating GFR, with high biases and unacceptably high errors.

## Key messages

In critically ill patients with early AKI:

• Urinary clearance of creatinine should not be used as a reference method when validating new methods for assessing kidney function

• Estimating equations for assessment of GFR perform poorly.

## Abbreviations

AKI: acute kidney injury; CG: Cockcroft-Gault equation; CKD-EPI: Chronic Kidney Disease Epidemiology Collaboration equation; CrCl: creatinine clearance; ^51^Cr-EDTA: chromium-ethylenediaminetetraacetic acid; eGFR; estimated GFR; GFR: glomerular filtration rate; GFR_51Cr-EDTA_: glomerular filtration rate measured with chromium-ethylenediaminetetraacetic acid infusion clearance; GFR_CG_: glomerular filtration rate estimated with the Cockcroft-Gault equation; GFR_CKD-EPI_: glomerular filtration rate estimated with Chronic Kidney Disease Epidemiology Collaboration equation; GFR_CrCl_: glomerular filtration rate estimated with urinary creatinine clearance; GFR_MDRD_: glomerular filtration rate estimated with the Modification of Diet in Renal Disease equation; ICU: intensive care unit; MDRD, Modification of Diet in Renal Disease equation.

## Competing interests

The authors declare that they have no competing interests.

## Authors' contributions

All authors participated in the study design. GB collected and prepared the data and performed the statistical analysis. GB and BR performed the experimental procedures. All authors participated in writing the paper and all read and approved the final manuscript.

## Supplementary Material

Additional file 1 **Supplementary Table 1**.. Click here for file
