# Supplementary material for: Assessing glomerular filtration rate (GFR) in critically ill patients with acute kidney injury - true GFR versus urinary creatinine clearance and estimating equations
Source: Crit Care. 2013 Jun 15;17(3):R108. doi: 10.1186/cc12777 (PMC4056314; doi:10.1186/cc12777)
Supplement: Additional file 1 — Supplementary Table 1.. [file cc12777-S1.DOCX]

**Supplementary Table 1**

**Formulae for estimating glomerular filtration rate.**

| **Measured 30 min urinary creatinine clearance:**  CrCl (mL/min/1.73m^2^) = (U-vol x U-creat x 1.73) / (S-creat x 30min x BSA)  BSA (m^2^) = weight (kg)^0.425^ x height (cm)^0.725^ x 71.84/10 000  **The Cockcroft-Gault equation:**  CrCl (mL/min/1.73 m^2^) = ([140-age] x weight) x 1.73 / (S-creat x 72) x BSA ( x 0.85 if female)  *Cockcroft-Gault adjusted for ideal body weigt (IBW):*  CrCl (mL/min/1.73 m^2^) = ([140-age] x IBW) x 1.73 / (S-creat x 72) x BSA ( x 0.85 if female)  *IBW (male)* = 50 kg + 0.9 kg for each cm > 150 cm in height  *IBW (female)* = 45 kg + 0.9 kg for each cm > 150 cm in height  **The simplified refitted MDRD equation:**  GFR (mL/min/1.73 m^2^) = 175 x S-creat ^-1.154^ x Age^-0.203^ ( x 0.742 if female) ( x 1.212 if black)  **The CKD-EPI equation:**  *Women:*  GFR (mL/min/1.73 m^2^) = 144 x (S-creat/0.7)^-0.329^ x 0.993^age^ ( x 1.15 if black) if S-creat ≤ 0.7 mg/dL  GFR (mL/min/1.73 m^2^) = 144 x (S-creat/0.7)^-1.209^ x 0.993^age^ ( x 1.15 if black) if S-creat > 0.7 mg/dL  *Men:*  GFR (mL/min/1.73 m^2^) = 141 x (S-creat/0.9)^-0.411^ x 0.993^age^ ( x 1.16 if black) if S-creat ≤ 0.9 mg/dL  GFR (mL/min/1.73 m^2^) = 141 x (S-creat/0.9)^-1.209^ x 0.993^age^ ( x 1.16 if black) if S-creat > 0.9 mg/dL |
| --- |

CrCl, urinary creatinine clearance; U-vol, urine volume; U-creat, urinary creatinine concentration; S-creat, serum creatinine concentration in mg/dL; BSA, body surface area; IBW, ideal body weight; MDRD, modification in diet in renal disease; GFR, glomerular filtration rate; CKD-EPI, chronic kidney disease epidemiology collaboration.
